# Defining the Legal Boundaries of Psychiatric Confidentiality: A Case Report

**DOI:** 10.7759/cureus.110794

**Published:** 2026-06-13

**Authors:** Carson Balen, Joseph Silverstein, Ryan Chi, Zayd Chishti, Amie Gerodimos

**Affiliations:** 1 Medicine, Morsani College of Medicine, University of South Florida, Tampa, USA; 2 Biological Sciences, University of California Los Angeles, Los Angeles, USA; 3 Psychiatry, Morsani College of Medicine, University of South Florida, Tampa, USA

**Keywords:** confidentiality, duty to warn, psychiatric caselaw, psychiatric disclosure, tarasoff, therapeutic privilege, violence

## Abstract

Psychiatry frequently requires clinicians to navigate the tension between therapeutic confidentiality and legal responsibility. Courts have historically recognized the importance of patient privacy in psychiatric treatment, yet landmark decisions have established circumstances in which clinicians may have a duty to warn identifiable victims when credible threats arise. Despite these legal frameworks, applying these principles in real clinical settings can be complex. We present a hospitalized patient admitted following a suicide attempt whose previous statements raised concern for potential violence toward another individual. The case highlights the uncertainty clinicians face when determining whether patient disclosures cross the legal threshold that may justify breaching psychiatric confidentiality.

## Introduction

Psychiatry sits at an intersection between clinical judgment and legal obligation. This overlap is especially apparent in high-stakes contexts involving suicide or homicide, where foreseeability warrants potential legal duty. In these settings, inadequate legal literacy increases the likelihood of preventable harm. Competent psychiatric care, therefore, requires both therapeutic skill and a working understanding of legal boundaries. 

The legal protection of patient-provider confidentiality primarily developed incrementally through case law. Though state and federal courts consistently recognized the value of confidentiality in a broader medical context, few such cases directly addressed the psychiatric setting specifically. An early such example, however, did arise in Binder v Ruvell (1952), where an Illinois court supported a psychiatrist’s refusal to disclose patient information based on the concept of therapeutic trust [1]. This precedent was later solidified decades later in Jaffee v. Redmond (1996). In this case, the prosecution attempted to use documentation obtained from a social worker to cross-examine a criminal defendant [2]. The Supreme Court rejected these efforts, recognizing that psychotherapy requires assurance of privacy to facilitate vulnerable communication [2]. The latter case remains monumental in establishing a federal standard for psychotherapist privilege. 

In a parallel series of cases, also addressing the therapeutic setting, courts delineated circumstances in which confidentiality demonstrated a potential to interfere with public safety. One such example arose in 1974 in Tarasoff v. Regents of the University of California, where a patient disclosed to a psychologist an intent to murder a woman; approximately two months later, he carried out the killing. Accordingly, the court imposed a duty to take reasonable steps when a patient makes a serious, credible threat toward an identifiable victim [3]. That precedent was refined in 1980, where Thompson v. The County of Alameda clarified that vague threats do not trigger such a duty to warn [4]. Across these decisions, psychiatrists were given a concrete legal structure to anchor and guide their clinical judgment.

In practice, psychiatrists must balance confidentiality, therapeutic alliance, and public protection. Confidentiality remains foundational because it enables effective treatment and preserves patient trust. However, when credible imminent threats toward identifiable individuals emerge, legal breaches are permissible in the interest of public safety. Against this backdrop, we present a patient involuntarily admitted following a suicide attempt and who subsequently made threats toward another individual during their hospitalization, raising this tension.

## Case presentation

A 67-year-old man with a past psychiatric history of alcohol/cocaine use disorder, major depressive disorder, and neuropathy was brought to a local behavioral hospital. The patient was involuntarily committed to a psychiatric hospital.

On the day of the incident, the patient contacted a close friend, requesting him to care for his dogs after he was gone. Concerned, this friend contacted emergency medical services, who found the patient unconscious secondary to a polysubstance overdose. The patient reported having ingested alprazolam, sedative hypnotics, and NSAIDs while simultaneously intoxicated with alcohol. A loaded firearm was found nearby, which was confiscated by law enforcement.

Laboratory findings confirmed the ingested substances reported by the patient, as shown in Table 1. Upon intake, special attention was made to elucidating the precipitating factors that led to the attempted overdose. Thorough history taking revealed that over the preceding six weeks, the patient reported insomnia, decreased appetite, and approximately thirty pounds of unintentional weight loss. Furthermore, the patient endorsed the presence of many stressors, including current unemployment, recent family challenges, and a financial dispute with a local mechanic. Regarding this last stressor, collateral obtained from the patient's primary care physician revealed that days before admission, the patient stated he would “kill his mechanic” or that he would be “in jail or dead.” Law enforcement was contacted at that time, though the patient was not detained.

**Table 1:** Urine Drug Screen on Admission POC: point of care; MDMA: 3,4-methylenedioxymethamphetamine

Parameter/drug tested	Result
POC barbiturates	Negative
POC benzodiazepine	Positive/abnormal
POC buprenorphine	Negative
POC cocaine	Negative
POC marijuana	Positive/abnormal
POC MDMA	Negative
POC methadone	Negative
POC amphetamine	Negative
POC morphine	Negative
POC opiates	Negative
POC oxycodone	Negative
POC phencyclidine	Negative
POC tricyclic antidepressants	Negative
POC fentanyl	Negative

Treatment was initiated by a multidisciplinary team, which prioritized medical optimization and therapeutic support. On admission, the patient’s home medications included quetiapine 25 mg nightly, gabapentin 1200 mg twice daily, and alprazolam 1 mg three times daily. The patient was also found to be taking lithium prescribed by his primary care physician. The medication was discontinued to streamline his psychopharmacologic regimen and due to a lack of indication; no formal diagnosis of bipolar disorder existed, nor evidence of an established relationship with a psychiatric provider. Alprazolam was stopped, and diazepam was initiated with a structured taper plan to limit dependence. Quetiapine and gabapentin were continued, given the patient reported therapeutic benefit and tolerability. Finally, bupropion XL 150 mg daily and valproate 250 mg BID were initiated with the hopes of addressing the patient’s recent relapse of depressive symptoms and emotional instability. 

Daily therapeutic sessions were held by the team to monitor progress and assess treatment effectiveness. During early hospitalization, the patient reported that he felt “great.” However, such reporting was incongruous with the patient's affect, and the patient simultaneously endorsed low self-esteem and mounting guilt. Additionally, priority was placed on further characterizing the patient's past homicidal remarks. Upon questioning, the patient acknowledged that the statements were driven by his immediate emotions and that he would instead “contact the police” regarding the financial dispute rather than confront the individual directly.

In the days following, nursing and staff documentation reflected decreased irritability and improved cooperation with care. With time, the patient more consistently attended group therapy and interacted appropriately with peers. In therapy sessions, the patient repeatedly denied ongoing suicidal or homicidal ideation and did not repeat previous threats. The patient reported improved sleep, increased appetite, and endorsed anticipation of future milestones. Such a shift in behavior matched with both staff therapeutic observations and objective psychiatric outcome measures. 

In the final days of admission, therapy documentation had tracked consistent improvements in mood. Additionally, the patient himself reported a positive response to his adjusted pharmacologic regimen. Taken together, these changes led the team to begin the process of discharge planning as the patient was no longer deemed a danger to himself or others by staff. The patient received instructions for outpatient therapy follow-up and guidance on obtaining his prescribed medications. He was discharged with appropriate social services and arranged transportation.

## Discussion

Confidentiality in psychiatry has a strong legal foundation and may only be breached under specific conditions. A series of legal proceedings has consistently necessitated credible and concrete threats to meet the threshold of disclosure. This standard partially stems from the belief that patient privacy is an essential aspect of effective care. While generally heralded in the broader clinical practice, confidentiality in the field of psychiatry has been shown to be uniquely tied to clinical outcomes and wider patient-provider interactions. However, when tangible threats to the greater public do arise, the potential for sustained treatment and preserved alliance must be weighed against perceived danger.

The therapeutic alliance is critical in the effective treatment of psychiatric patients. As such, clinicians are obligated to make their best efforts to preserve these relations. Empirical data suggest that the therapeutic alliance is a major determinant of attrition. In one meta-analysis of child and adolescent psychotherapy, researchers found alliance variables accounted for approximately 7% of outcome variance and were among the strongest predictors of dropout [5]. Beyond retention, alliance may also serve as a direct predictor of clinical outcomes. One systematic review of 19 studies examining suicidal experiences found that a stronger therapeutic alliance was consistently associated with reductions in suicidal ideation and attempts [6]. In a separate meta-analysis of 295 studies involving over 30,000 patients, researchers found that rated alliance accounted for roughly 8% of outcome variance [7]. These findings suggest that patient-provider relations may directly support adherence while simultaneously having a tangible effect on patient outcomes. In our patient, potential disclosure was balanced against the erosion of clinical rapport, which would possibly compromise both engagement/retention and the effectiveness of interventions.

Simultaneously, the clinical objective in our case extended to modifying violent risk through techniques shown to reduce recidivism and aggression. More specifically, patients prone to violent behavior have historically responded to therapeutic intervention in studied settings. A meta-analysis of cognitive behavioral therapy (CBT) programs for offenders demonstrated a mean reduction in recidivism of approximately 25% [8]. Another systematic review of prison-based psychological interventions found a pooled odds ratio of 0.72 for reoffending among treated individuals compared with controls [9]. Pharmacologic evidence is similarly strong. One cohort study of over 80,000 patients found that periods of antipsychotic treatment were associated with a 45% reduction in violent crime risk, and periods of mood stabilizer treatment were associated with a 24% reduction in violent crime risk [10]. These analyses indicate that violence risk may be a modifiable and treatable quality. Tying back to our patient, the initiation of valproate with concurrent implementation of cognitive behavioral techniques constituted multimodal efforts by the treatment team to reduce aggression risk. 

Ultimately, the treating providers determined that breaching psychiatric confidentiality was not warranted. Our patient’s homicidal statements lacked imminence and an actionable plan, which placed them below legal thresholds for mandatory disclosure. The decision was evidence-based, with many studies suggesting that alliance disruption may increase dropout rates and ultimately lead to poorer clinical outcomes. Additionally, treatments specifically associated with reduced violence were being actively pursued. In this case, confidentiality preserved engagement, allowed continued risk assessment, and enabled effective delivery of psychiatric interventions.

## Conclusions

This case illustrates the boundary between legal duty and clinical responsibility. Confidentiality is a tangible tool that can improve both patient trust and outcomes. Breaching this principle requires meeting a legally justifiable standard. In our patient, the threats made were temporally remote and without a specific articulated plan, and so were not deemed to meet the threshold of mandatory reporting. The restraint in this decision allowed for a preserved therapeutic alliance and eventual effective treatment and discharge of a mentally vulnerable patient.
